# PARP-1 Modulates Amyloid Beta Peptide-Induced Neuronal Damage

**DOI:** 10.1371/journal.pone.0072169

**Published:** 2013-09-24

**Authors:** Sara Martire, Andrea Fuso, Dante Rotili, Italo Tempera, Cesare Giordano, Ivana De Zottis, Alessia Muzi, Patrizia Vernole, Grazia Graziani, Emanuela Lococo, Martina Faraldi, Bruno Maras, Sigfrido Scarpa, Luciana Mosca, Maria d'Erme

**Affiliations:** 1 Department of Biochemical Sciences, Sapienza University, Rome, Italy; 2 Department of Psychology-Sec.Neuroscience, Sapienza University, Rome, Italy; 3 Department of Pharmaceutical Studies, Sapienza University, Rome, Italy; 4 Fels Institute for Cancer Research & Molecular Biology, Temple University, Philadelphia, Pennsylvania, United States of America; 5 Department of Neuroscience, University of Roma “Tor Vergata”, Rome, Italy; 6 Department of Public Health and Cell Biology, University of Roma “Tor Vergata”, Rome, Italy; 7 Department of Experimental Medicine, Sapienza University, Rome, Italy; 8 Department of Surgery “P.Valdoni”, Sapienza University, Rome, Italy; 9 Instituto Pasteur Fondazione Cenci Bolognetti, Sapienza University, Rome, Italy; University of Minnesota, United States of America

## Abstract

Amyloid beta peptide (Aβ) causes neurodegeneration by several mechanisms including oxidative stress, which is known to induce DNA damage with the consequent activation of poly (ADP-ribose) polymerase (PARP-1). To elucidate the role of PARP-1 in the neurodegenerative process, SH-SY5Y neuroblastoma cells were treated with Aβ_25–35_ fragment in the presence or absence of MC2050, a new PARP-1 inhibitor. Aβ_25–35_ induces an enhancement of PARP activity which is prevented by cell pre-treatment with MC2050. These data were confirmed by measuring PARP-1 activity in CHO cells transfected with amylod precursor protein and *in vivo* in brains specimens of TgCRND8 transgenic mice overproducing the amyloid peptide. Following Aβ_25–35_ exposure a significant increase in intracellular ROS was observed. These data were supported by the finding that Aβ_25–35_ induces DNA damage which in turn activates PARP-1. Challenge with Aβ_25–35_ is also able to activate NF-kB *via* PARP-1, as demonstrated by NF-kB impairment upon MC2050 treatment. Moreover, Aβ_25–35_ *via* PARP-1 induces a significant increase in the p53 protein level and a parallel decrease in the anti-apoptotic Bcl-2 protein. These overall data support the hypothesis of PARP-1 involvment in cellular responses induced by Aβ and hence a possible rationale for the implication of PARP-1 in neurodegeneration is discussed.

## Introduction

Free radical damage, which occurs during oxidative stress, is associated with neurodegenerative disorders, such as Alzheimer's disease (AD) and Parkinson's disease (PD) [1], [2]. The major cause of free radicals overproduction seems to be related to the accumulation of misfolded protein aggregates in brain tissues. In AD, the major component of these protein aggregates present in senile plaques, is the amyloid beta (Aβ), a peptide of 39–42 amino acid residues which derives from the sequential proteolytic processing of the amyloid precursor protein (APP) by beta- and gamma-secretases. When an unbalance between Aβ production and clearance due to genetic and/or environmental factors occurs, Aβ oligomerization takes places producing different species of soluble supramolecular assemblies and some of them finally converge towards fibrillar formation [3], [4]. Aβ plays a central role in the pathogenesis of AD, by causing neurodegeneration and disrupting the cognitive function although the molecular pathways leading to neuronal impairment are not yet fully elucidated. It has been shown that early formed pre-fibrillar aggregates of Aβ are mainly endowed with cytotoxicity, whereas mature fibrils are much less toxic or even harmless [5]. In particular, soluble Aβ oligomers are associated with the generation of free radicals *via* direct and indirect mechanisms: in the direct one, Aβ binds to transition metals ions, acquiring an oxidase activity leading to hydrogen peroxide production [6]. In the indirect mechanism neurons or microglia stimulated by Aβ oligomers produce free oxygen radicals by activation of NADPH oxidase [7].

Free radical injury may be responsible for neuronal loss by inducing DNA damage that in turn activates poly (ADP-ribose) polymerase enzyme (PARP-1).

PARP-1 is a 116 kDa zinc-binding nuclear enzyme consisting of three main domains: the N-terminal DNA-binding domain containing two zinc fingers motifs, the automodification domain, and the C-terminal catalytic domain. This enzyme catalyzes the covalent addition of the ADP-ribose moiety of nicotinamide adenine dinucleotide (NAD^+^) to nuclear proteins including histones, transcription factors and PARP-1 itself, and the subsequent elongation of the polymer. PARP-1 is involved in many physiological processes such as gene expression, maintenance of genomic stability and cell death and differentiation [8] [9].

Extensive PARP-1 activation by DNA damage contributes to the development and progression of various chronic diseases including diabetes, cancer, viral infections and neurodegenerative diseases [10]–[15]. In particular, the findings that parkinsonian neurotoxins and Aβ activate PARP-1 in dopaminergic neurons and hippocampal slices respectively, suggest a relationship between PARP-1 and neurodegeneration [16]–[18].

In the present study we focused on the comprehension of the molecular mechanisms that lead to PARP-1 activation by Aβ in SH-SY5Y neuroblastoma derived cells and in transgenic mice TgCRND8, an early onset model of AD and to the downstream ways activated by PARP-1. Since the suppression of over-activated PARP-1 by specific inhibitors might represent a useful tool to prevent neurotoxicity, we also analyzed the protection of SH-SY5Y cells from Aβ harmful effects by a newly synthesized PARP-1 inhibitor, MC2050 [19]. Our data show that challenge of SH-SY5Y cells with Aβ significantly increased PARP-1 activity following ROS generation and DNA damage and PARP-1 activated NF-kB and modulates pro-apoptotic proteins. These effects were significantly decreased in the presence of MC2050 suggesting a potential therapeutic application for this compound in neurodegenerative disease.

## Materials and Methods

### Preparation of the test substances

MC2050 (2-[2-(4-(2-pyridyl)-1-piperazinyl) ethylsulfanyl]-3*H*-quinazolin-4-one) dihydrochloride, a new PARP inhibitor was dissolved in water at an initial concentration of 50 mM and then diluted to a final concentration of 50 µM in PBS or culture medium. Two other PARP inhibitors were used in this work: 3-ABA (3-aminobenzamide) and PJ34. 3-ABA was dissolved at a concentration of 4 M in DMSO and then diluted to a final concentration of 4 mM in culture medium; PJ34 was dissolved at a concentration of 50 mM in H_2_O and then diluted to a final concentration of 5 µM in culture medium. Amyloid beta peptide 25–35 fragment (Aβ_25–35_) synthesized by conventional solid phase chemistry [20], was aggregated overnight at 37°C in phosphate buffered saline at a concentration of 1mM.

### Animals

TgCRND8 mice (TgCRND8–129Sv), carrying the double mutant form APP695 (KM670/671NL+V717F), were obtained from Dr. David Westaway – Center of Research on Neurodegenerative Disease – University of Toronto and maintained in the heterozygous status (TgCRND8+/−) by mating male Tg with female WT (TgCRND8−/−) 129Sv mice (Charles River Laboratories, Wilmington MA, USA). These mice show amyloid peptide deposition at 3 months, with dense-cored plaques and neuritic pathology evident from 5 months of age [21].

### Mice treatment

Biochemical analyses were carried out on a total of 16 animals (8 WT and 8 Tg). At the third month of life mice were anesthetized and sacrificed to obtain brains. Brains were perfused with PBS, removed and bisected on the sagittal plane. Hippocampus and entorhinal cortex, sites of major pathological changes in AD, were stored at −80°C and used for the biochemical and molecular analyses.

All experiments were performed in order to sacrifice the minimum number of animals required and were previously approved by author's Institution according to guidelines of the Italian Ministry of Health (D.L. 92/116) and of the EC directives 86/609/EEC and 2010/63/EU on the protection of animals used for experimental and other scientific purposes. According to Italian laws on animal care, the experimental protocol for the experiments described in this paper has been “communicated” to the Ministry of Health (IACUC) without necessity of any formal approval number.

### Cell treatment

The human dopaminergic neuroblastoma cell line SH-SY5Y was obtained by ICLC (Genova, Italy) and maintained in DMEM/F12 supplemented with 10% heat-inactivated foetal bovine serum and 2 mM glutamine, at 37°C and under 5% CO_2_ in a humidified incubator. SH-SY5Y, an extremely versatile neuronal cell line, has been extensively utilized as an experimental model for the study of neuronal death processes induced by several agents [22]. Cells were treated with 10 µM Aβ_25–35_ in the presence or absence of the PARP-1 inhibitors. The inhibitors were added before Aβ_25–35_ treatment. In some experiments SH-SY5Y were also pre-treated with 5 µM quercetin, a natural antioxidant agent.

7PA2, a Chinese hamster ovary (CHO) cell line stably transfected with a complementary DNA coding for APP751 containing the Val717Phe familial Alzheimer's disease mutation that leads to Aβ overproduction, was a kind gift of Prof. D. Walsh, USA. CHO cells were used as a control. Both cell lines were maintained in DMEM/F12 supplemented with 10% heat-inactivated foetal bovine serum and 2 mM glutamine, at 37°C and under 5% CO_2_ in a humidified incubator.

### PARP-1 activity assay

PARP-1 activity was tested in SH-SY5Y cells, in 7PA2 cells and in mice with a colorimetric PARP assay Kit from Trevigen. Briefly, the assay measures the incorporation of biotinylated poly (ADP-ribose) onto H1 histone in a 96 well plate. Enzyme assay was performed by adding biotinylated NAD^+^ to a reaction mixture containing lysates (20 µg protein). Cultured cells and homogenized tissue from hippocampus and entorhinal cortex were collected and centrifuged at 400 *g* for 10 min at 4°C and then resuspended in 100 μl of PARP lysis buffer (PARP buffer, 0.4M NaCl, 1% NP-40, 0.4 M PMSF, protease inhibitor). The lysates were incubated on ice for 30 min and centrifuged at 10000 *g* for 10 min at 4°C. The collected supernatants were subjected to protein determination by Bradford Assay (BioRAD). PARP Cocktail (containing biotinylated NAD^+^) was added to each wells and incubated for 60 min. Then, diluted Strep-HRP was added to each well, incubated for 20 min at room temperature and followed by TACS-Sapphire incubation for additional 30 min in the dark. The reaction was stopped by adding 0.2 M HCl and then the plate was read at 450 nm.

### Cell viability assay

Cell viability was assessed by using the dye [4,5-dimethylthiazol-2-yl]-2,5-diphenyltetrazolium bromide (MTT). The assay is based on the ability of living cells to convert MTT into an insoluble purple-coloured formazan, whose amount is proportional to the number of living cells. Cells seeded in 96 wells plates at a density of 15000 cells/well were exposed to Aβ_25–35_ at a final concentration of 10 µM. Cell viability was assessed at 24, 48 and 72 h. After the indicated time intervals, cells were treated with 20 µl of a 5 mg/ml solution of MTT in PBS and incubated at 37°C for 2 h. After discarding the medium, the formazan was extracted with DMSO, and absorbance read at 577 nm with a reference at 690 nm.

### Cell Cycle

The SH-SY5Y cells were treated with Aβ_25–35_ (10 μM) for 24 and 48 h. The cells were then washed with PBS and centrifuged at 900 *g* for 10 min, resuspended in 70% ethanol and then centrifuged. The pellet was resuspended at 37°C for 30 min in 1 ml of PBS containing 100 μg of propidium iodide and 100 μg of RNAse. The samples were then analyzed by EPICS XL-MCL Flow cytometer (Coulter).

### Permeation of the membrane

To assess the ability of amyloid peptide to alter the permeability of SH-SY5Y membrane, cells seeded in 96 wells plates at a density of 13000 cells/well were treated with Aβ_25–35_10 μM, in the presence or absence of MC2050. At different times the medium was removed and the cells were resuspended in 100 μl of PBS containing 1 μM Sytox Green. After Aβ_25–35_ addition, the increase in fluorescence, due to the binding of the dye to intracellular DNA, was measured at 37°C by a Polarstar Galaxy microplate reader (BMG Labotechnologies, Offenburg, Germany) using 485 and 520 nm filters for excitation and emission, respectively.

### ROS detection

Reactive oxygen species formation in SH-SY5Y cells treated with Aβ_25–35_ was assayed by flow cytometry using the dye DCF-DA (dichlorofluoresceine-diacetate) and following standard methods. Briefly, DCF-DA (final concentration 10 µM) was added to cells grown on 6 wells plates for 15 min at 37°C in a humidified incubator and then cells were treated with the indicated amount of Aβ_25–35_, in the presence or absence of 5 µM quercetin, at different times. Cells were then scraped, washed in PBS and analyzed by Epics XL-MCL flow cytometer (Coulter) equipped with an Argon laser at 488 nm. Cells were gated on the basis of forward-angle light-scatter (FS) and 90° light-scatter parameters (SS). For every histogram, a minimum of 10000 events were counted. The mean fluorescence intensity was analyzed and expressed as percentage of relative ROS level *vs.* control cells.

### Comet assay

Oxidative DNA damage was studied by electrophoresis of single cell in agarose gel.

SH-SY5Y (30000 cells) were resuspended in a 0,65% of low melting point agarose (LMPA) and left in a water bath (37°C) and then smeared on slides, which were earlier coated with a thin layer of 0.65% LMPA and left to dry out. After 10 min the slides were placed in the lysis buffer (2.5 M NaCl, 100 mM EDTA, 10 mM Tris, pH 10, 1% Triton X–100 and 10% DMSO) for 1 h at 4°C in dark. The slides were left for 40 min in a buffer for electrophoresis (1 mM EDTA, 300 mM NaOH pH >13) at 4°C in order to lose nuclear super-coiled DNA. Then, they were subjected to low-voltage electrophoresis at 300 mA, for 30 min at 4°C, and washed three times with neutral cold buffer (0.4 M Tris/HCl, pH 7.5). The slides were finally submerged for 5 min in distilled water and stained with propidium iodide at concentration of 2.5 μg/ml. DNA damage was visualized by an epifluorescence microscope (Olympus IX-50) equipped with a fluorescence source. The analysis of oxidative DNA damages was determined by olive moment parameter using the COMET PLUS 2.9 software (Comet Plus, Theta System Gmbh, Germany).

### Electrophoretic mobility shift assay (EMSA)

SH-SY5Y cells were resuspended in high saline buffer (50 mM Tris-HCl, 400 nM NaCl, 1 mM EDTA, 1% Triton, 0.5% NP-40, 10% Glycerol, 2 mM DTT), protease and phosphatases inhibitors. The resulting cell lysates were centrifuged for 5 min at 15000 *g* at 4°C and 20 μg of protein extract were incubated with binding buffer 2X (20 mM Tris-HCl, 2 mM EDTA, 10% glycerol), 1mg/mL BSA, 1mg/mL Poly-d (I-C), 15 fmol/µL DIG-labeled (DIG oligonucleotide 3′ end-labeling kit, Roche Applied Science) NF–kB DNA probe [23]. The sequence of the “KB” oligonucleotide used was as follows:

5′- AGCTTCAGAGGGGACTTTCCGAGAGG –3′

3′- AGTCTCCCCTGAAAGGCTCTCCAGCT –5′

The DNA-protein complex formed was separated on 4% native polyacrylamide gel and transferred onto nitrocellulose membrane o/n. Detection was performed by DIG Gel Shift Kit (Roche^®^). Supershift assay using NF-κB p65 and p50 antibodies (Millipore) was also carried out to confirm the specificity of NF-κB DNA-binding activity. 30 μg of proteins from each sample were subjected to Western blot to verify equal loading.

### Subcellular fractionation

SH-SY5Y cells were suspended in hypotonic buffer (10 mM HEPES, 1.5 mM MgCl_2_, 10 mM KCl, 1 mM PMSF, protease inhibitors, 10 mM DTT, 0.1% NP-40) and incubated for 30 min on ice. The cell lysate was centrifuged for 5 min at 4500 *g* at 4°C, and the supernatant collected (cytosolic fraction). The nuclear fraction was obtained by sonication of the pellet in hypertonic buffer (5 mM HEPES, 1,5 mM MgCl_2_, 0,2 mM EDTA, 0,5 mM DTT, 26% glycerol, 1 mM PMSF, protease inhibitors), mixed quickly and incubated for 1 h on ice. After centrifugation for 20 min at 10000 *g* the supernatant was collected. Hence, cytosolic and nuclear fractions were submitted to western blotting analyses.

### Western blotting

SH-SY5Y cells and mice brain tissues were lysed in 80 μl RIPA buffer (50 mM Tris-HCl pH 7.4, 150 mM NaCl, 0,1% NP-40, 0.1% SDS, 1 mM EDTA, 1 mM Na_3_VO_4_, 1 mM PMSF, 0,5 NaDOC, protease inhibitors). The lysates were incubated on ice for 30 min and centrifuged at 10000 *g* for 10 min at 4°C. The supernatants were collected and proteins quantification were performed using a Bradford Assay (BIO-RAD). Equal amounts of protein were separated on 12% SDS-PAGE Criterion (BIO-RAD) and subsequently transferred to nitrocellulose membrane (GE Healthcare). The membrane was probed with the primary anti-PAR (1∶5000) (Alexis), anti-PARP (1∶1000) (Alexis), anti-p53 (1∶400) (Sigma), anti-Bcl-2 (1:500) (Sigma), anti-p50 (1∶3000) (Millipore), anti-p65 (1∶1000) (Millipore), anti-actin (1∶5000) (Alexis), anti-vinculin (1∶500) (Santa Cruz), anti-lamin A/C (1∶1000) (Millipore) in PBS at 4°C overnight. The subsequent steps were performed with innovative SNAP i. d. system (Millipore). The bands were visualized by ECL system (Millipore) according to the manufacture's instructions. Densitometric analyses were performed with ImageJ software, and normalized to a reference protein.

### Statistical analysis

Results are expressed as mean ± SD for at least three separate experiments. Graphics and data analysis were performed using GraphPAD prism 4 software. Statistical analyses were performed using the ANOVA and Bonferroni post hoc test. P<0.05 was deemed significant.

## Results

### Aβ affects PARP activity

**Figure 1:**
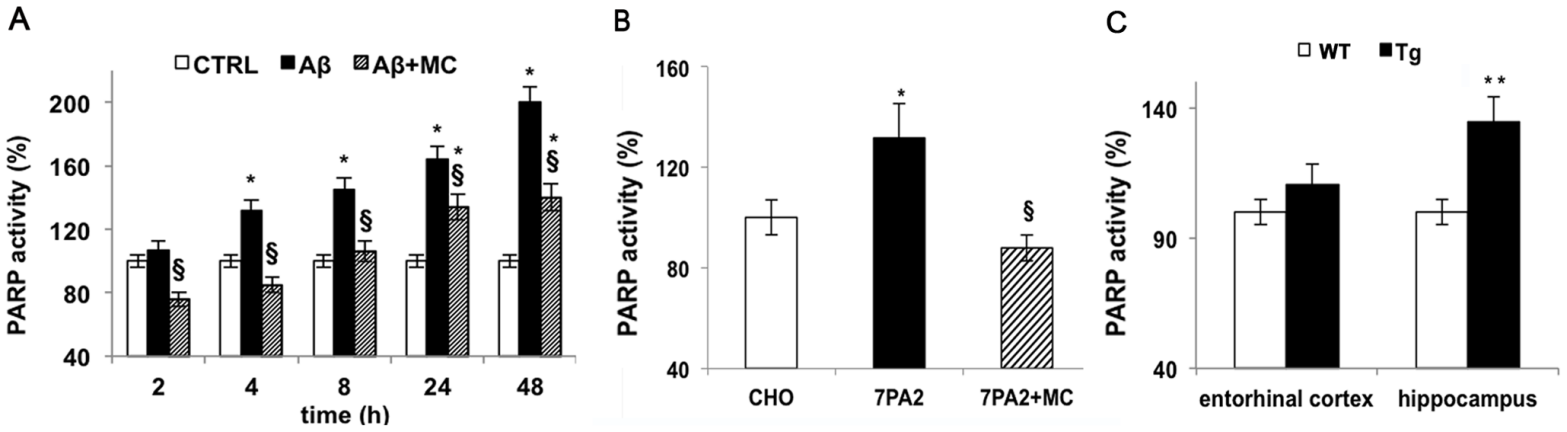
Aβ induces PARP-1 activation. PARP-1 activity was determined in cells and tissues homogenates by the use of a dedicated colorimetric Kit from Trevigen, following manufacturer instructions. A) SH-SY5Y cells treated with 10 µM Aβ_25–35_ in the presence or absence of 50 µM MC2050 for the indicated times (white bar  =  control cells; black bar  =  Aβ_25–35_ treated cells; stripped bar  =  cells treated with Aβ_25–35_ in the presence of MC2050); B) PARP-1 activity in 7PA2 cells in the presence or absence of 50 µM MC2050. CHO cells were used as control (white bar  =  control cells; black bar  = 7PA2 cells; stripped bar  =  7PA2 cells in the presence of MC2050); C) Entorhinal cortex and hippocampus of transgenic mice TgCRND8 (Tg  =  black bar) compared to wild type mice 129Sv (WT  =  white bar). The values are the mean ± S.D. of at least three independent experiments. *p<0.05 *vs*. control cells or WT; ^§^ *p*<0.05 *vs*. Aβ_25–35_-treated cells or 7PA2 cells.

To investigate whether Aβ could induce an increase in PARP-1 activity, SH-SY5Y cells were treated Aβ_25–35_, a toxic fragment of the full length Aβ_1–42_, for 2, 4, 8 and 24 h and enzymatic activity was assayed by a dedicated colorimetric kit. Results, reported in Fig. 1A, show that the amyloid peptide increased PARP-1 activity in a time-dependent manner. After 24 h, the activity in treated cells was 65% higher than in control. This activity level was maintained even when the stimulation was prolonged up to 48 h. Cells pre-treatment with 50 µM MC2050 prevented Aβ_25–35_ induced PARP-1 activation. The same results were obtained using 3 mM 3-ABA, a well known PARP inhibitor (data not shown). To assess whether the full length fragment Aβ_1–42_ is able to exert PARP-1 activation, 7PA2, CHO cells transfected with a mutated APP that lead to amyloid peptide overproduction, were assayed for PARP-1 activity. A 40% increase was observed in 7PA2 with respect to control CHO cells (Fig. 1B).

This demonstrates that a constant activation of PARP-1 takes place in the presence of both amyloid peptide species allowing us to use Aβ_25–35_ for the subsequent set of experiments since it is more easy-to-handle, cheaper and the results are more reproducible.

**Figure 2:**
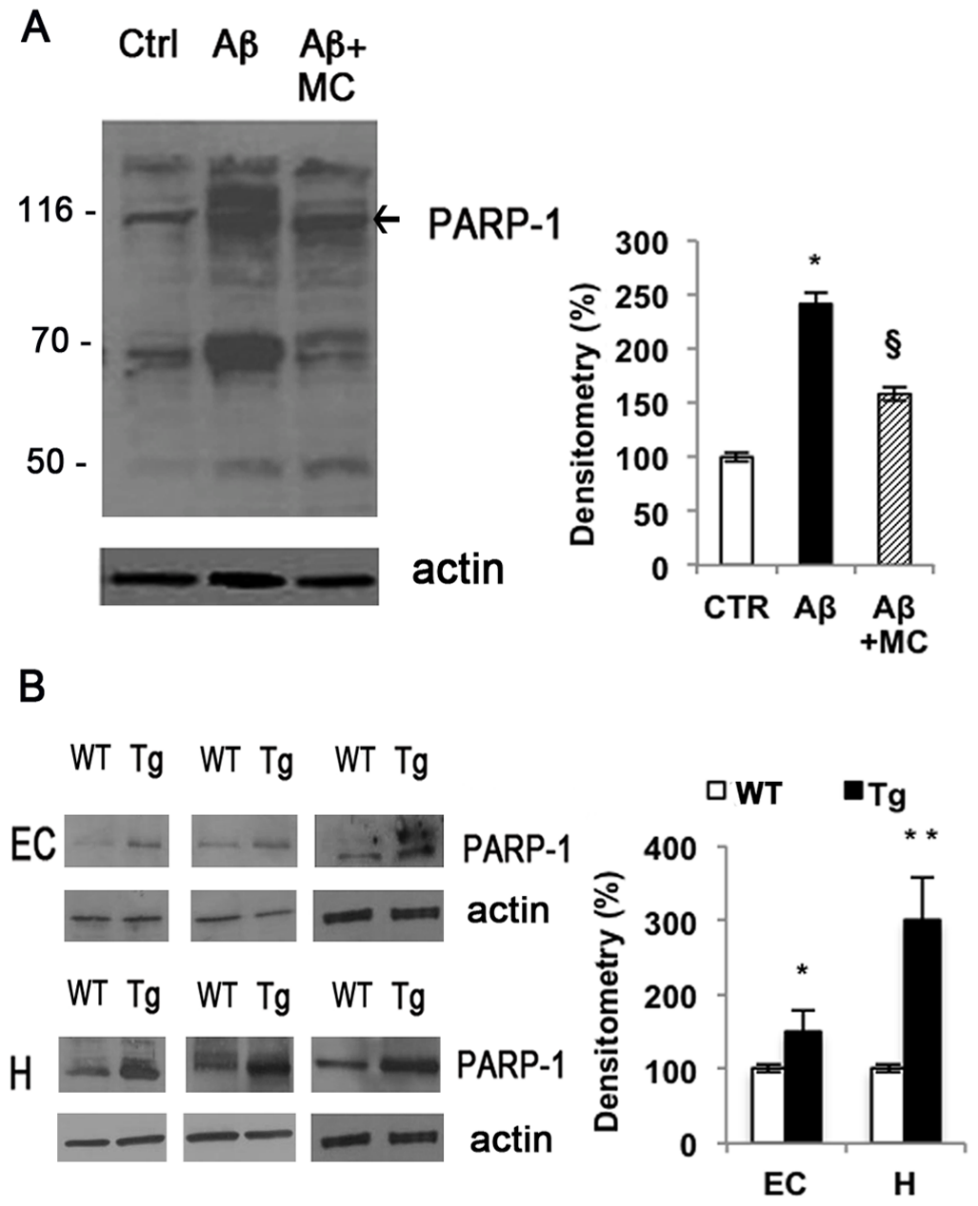
Aβ induces protein PARylation. Equal concentrations of proteins (30 µg) from SH-SY5Y cell lysates treated with 10µM Aβ_25–35_ for 4 h in the presence or absence of 50 µM MC2050 (A) (white bar  =  control cells; black bar  =  Aβ_25–35_ treated cells; stripped bar  =  cells treated with Aβ_25–35_ in the presence of MC2050) and from entorhinal cortex and hippocampus of wild type and transgenic mice (B) (white bar  =  Wild Type mice; black bar  =  Transgenic mice) were subjected to 12% SDS-PAGE, and immunoblotted with anti-PAR polymer (1∶5000 Alexis). Densitometric analyses were performed with ImageJ software, and normalized to β-actin. Data are reported as percentage of control values and are the mean ± S.D. of at least three independent experiments. *p<0.05 *vs*. control cells or WT; **p<0.01 *vs*. WT; ^§^ *p<*0.05 *vs*. Aβ_25–35_-treated cells.

We confirmed a tight correlation between Aβ_25–35_ accumulation and PARP-1 activity by *in vivo* experiments using heterozygous transgenic mice. After 3 months, when early amyloid deposit occurs, mice were sacrificed and PARP-1 activity was assayed in hippocampus and in cortex compartments of both wild type and mutant mice. We observed an increase of more than 30% activity in the hippocampus, while a slight increase was recorded in the cortex region according to the direction of the spread of amyloid accumulation in AD (Fig. 1C). Moreover, immunoblot with anti-PAR antibodies showed an increase in PARylated proteins both in cells treated with Aβ_25–35_ up to 4 h, and in TgCRND8 mice. The pre-treatment of cells with MC2050 significantly reversed PARP-1 activation [Fig. 2 A, B].

These *in vitro* and *in vivo* results drove us to understand the molecular causes of this enhancement of PARP-1 activity in injured SH-SY5Y cells.

### Aβ_25–35_ generates membrane perturbation, ROS production and DNA damage

**Figure 3:**
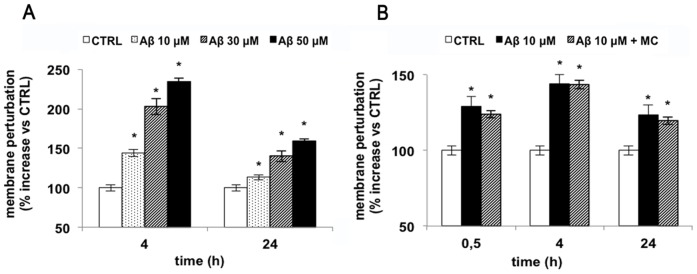
Aβ_25–35_ induces membrane perturbation in SH-SY5Y cells. 13000 cells were seeded in 96 well plates and treated with various amount of Aβ_25–35_ were incubated with 1 μM SYTOX™ Green in PBS. Changes in fluorescence were monitored (λex = 485 nm, λem  = 520 nm) and plotted as the percentage of fluorescence relative to the control cells. A) Time-course of cells treated with 10 µM Aβ_25–35_ (white bar  =  control cells; black bar  =  Aβ_25–35_ treated cells; stripped bar  =  cells treated with Aβ_25–35_ in the presence of MC2050); B) Dose-dependent effect of Aβ_25–35_ on membrane perturbation (white bar  =  control cells; dotted bar  = 10 µM Aβ_25–35_; stripped bar  = 30 µM Aβ_25–35_; black bar  = 50 µM Aβ_25–35_). Results are the mean of at least three independent experiments ± S.D. *p<0.05 *vs*. control cells.

Since several works demonstrated that the toxicity of amyloid oligomers and larger fibrillar aggregates is related to a perturbing effect on the biological function of the adjacent cellular membrane [24], [25], firstly we assayed the Aβ_25–35_ capability to induce membrane damage. Obtained data showed that a perturbation occurred when cells were treated with 10 μM Aβ up to 4 h (Fig. 3A) and that the extent of membrane perturbation is correlated with the amyloid concentration (Fig. 3B). We also treated the cells with amyloid peptide in the presence or absence of MC2050 to demonstrate whether this compound was able to prevent membrane perturbation. MC2050 did not influence the process, indicating that membrane damage was not mediated by PARP-1 activity (Fig. 3A).

**Figure 4:**
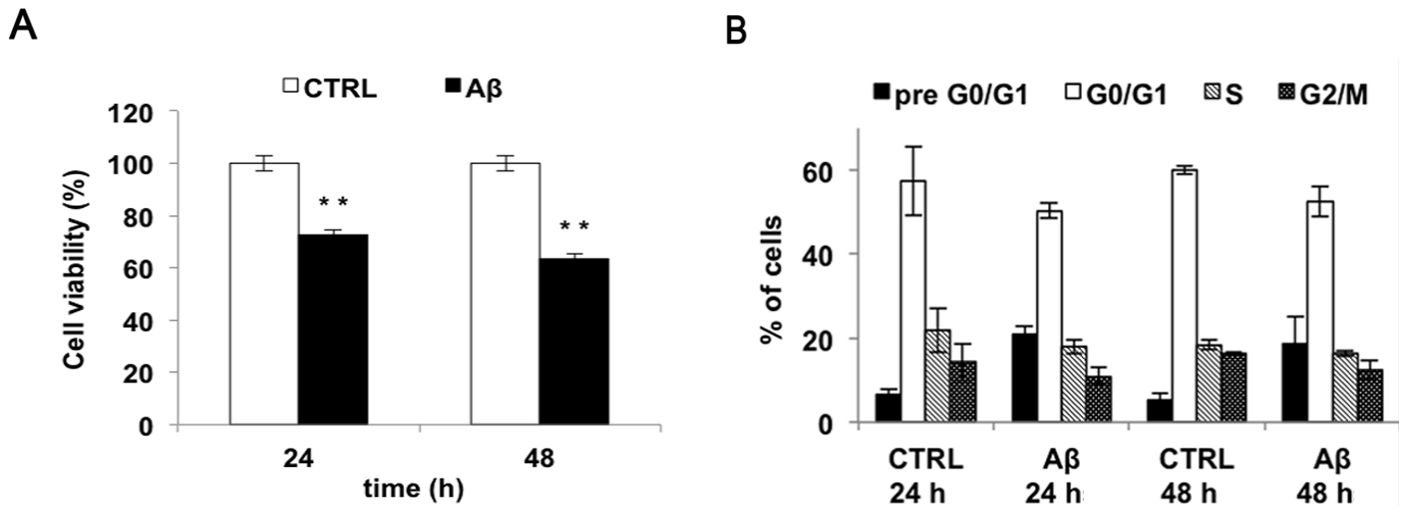
Effect of Aβ_25–35_ on SH-SY5Y neuroblastoma cell line viability. **A**) Cell viability of SH-SY5Y cells treated with 10 µM Aβ_25–35_ for 24, 48 h was monitored using MTT assay (white bar  =  control cells; black bar  =  Aβ_25–35_ treated cells). B) Cell cycle phases distribution of SH-SY5Y cells, as determined by FACS analysis Results are the mean of at least three independent experiments ± S.D. **p<0.01 *vs*. control cells.

In addition, to assess the cytotoxic effects exerted by Aβ_25–35_ on cells, viability assays were performed and the results revealed that the challenge with Aβ_25–35_ reduced cell viability in a time-dependent manner (Fig. 4A). To confirm these data we further checked cell toxicity by evaluating cell cycle after Aβ_25–35_ treatment. Results showed that the subG0 peak is significantly increased compared to control cells, raising from a basal value of about 4% to 20% within 48 h (Fig. 4B). Preliminary experiments on primary neuron treated with amyloid peptide showed a similar toxic effect (data not shown).

**Figure 5:**
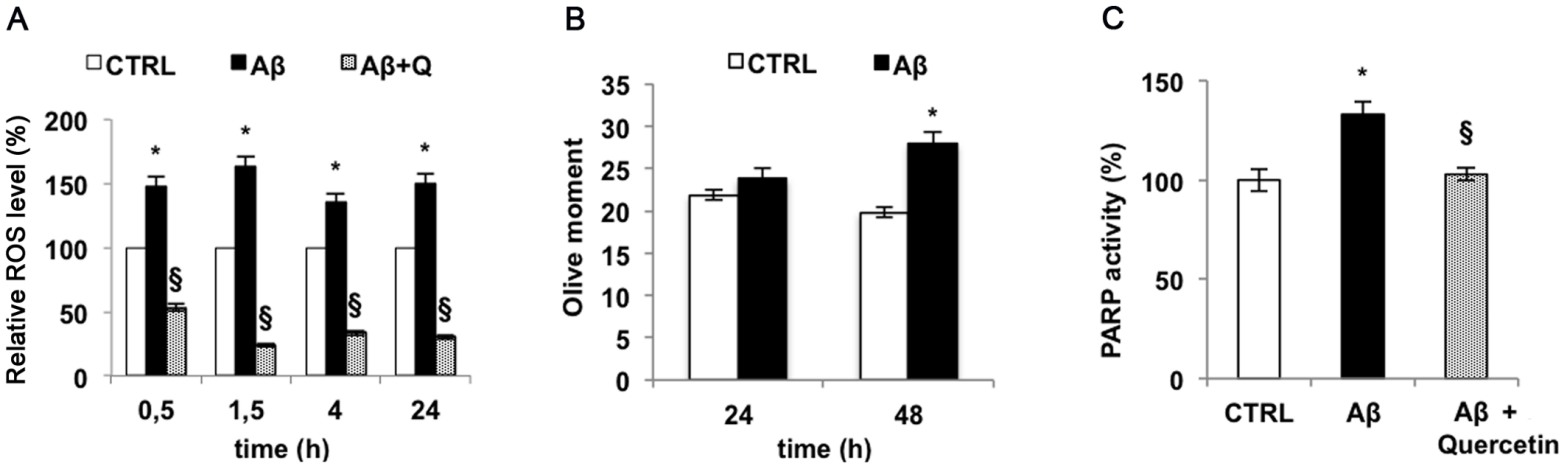
Aβ_25–35_ induces oxidative stress and DNA damage. A) ROS produced by SH-SY5Y cells treated with 10 µM Aβ_25–35_ at different times were revealed by flow cytometry using DCF-DA (white bar  =  control cells; black bar  =  Aβ_25–35_ treated cells; fine dotted bar  =  cells treated with Aβ_25–35_ in the presence of quercetin). B) DNA damage was evaluated by Comet assay and expressed as Olive moment in SH-SY5Y treated with 10 µM Aβ_25–35_ for 24 and 48 h (white bar  =  control cells; black bar  =  Aβ_25–35_ treated cells). C) PARP-1 activity in SH-SY5Y treated with 10 µM Aβ_25–35_ in the presence or absence of 5 µM quercetin by the use of a dedicated colorimetric Kit from Trevigen (white bar  =  control cells; black bar  =  Aβ_25–35_ treated cells; fine dotted bar  =  cells treated with Aβ_25–35_ in the presence of quercetin). Results are the mean of at least three independent experiments ± S.D. *p<0.05 *vs.* control cells; ^§^ *p*<0.05 *vs.* Aβ_25–35_-treated cells.

Finally, since a well-known potent inducer of PARP-1 activation is DNA damage caused by ROS, intracellular free radical production and DNA damage in SH-SY5Y cells challenged by Aβ_25–35_ were monitored. ROS production, determined by flow-cytometric analysis, was markedly increased up to 70–80% upon treatment with Aβ_25–35_ within the first 2 h with respect to the control and then gradually decreased to 40–50% at 24 h (Fig. 5 A). DNA damage was evaluated by Comet assay analysis (expressed as Olive moment), in SH-SY5Y cells treated for 24 h and 48 h with Aβ_25–35_ (Fig. 5 B). Our results showed that as a consequence of treatment with the amyloidogenic peptide, the increase of DNA damage was observed in SH-SY5Y cells.

Our data confirm the negative effect of Aβ_25–35_ on DNA integrity via ROS production and strongly suggest that PARP-1 activation depends on this event. To further strengthen this hypothesis, we used quercetin, a plant derived flavonoid, endowed with strong antioxidant capacity in order to blunt Aβ_25–35_ induced ROS production. When quercetin was added to the medium of SH-SY5Y cell cultures in the presence of Aβ_25–35_ the overall amount of free radicals dropped below the control level and PARP-1 activity did not show any variation with respect to the control (Fig. 5 A, C) strongly indicating that PARP-1 activation observed in Aβ_25–35_-treated cells depended on DNA damage caused by redox unbalance.

Then we moved on to investigate whether PARP-1 activation could affect cell signalling and cell death.

### MC2050 prevents activation of NF-κB induced by Aβ_25-35_

**Figure 6:**
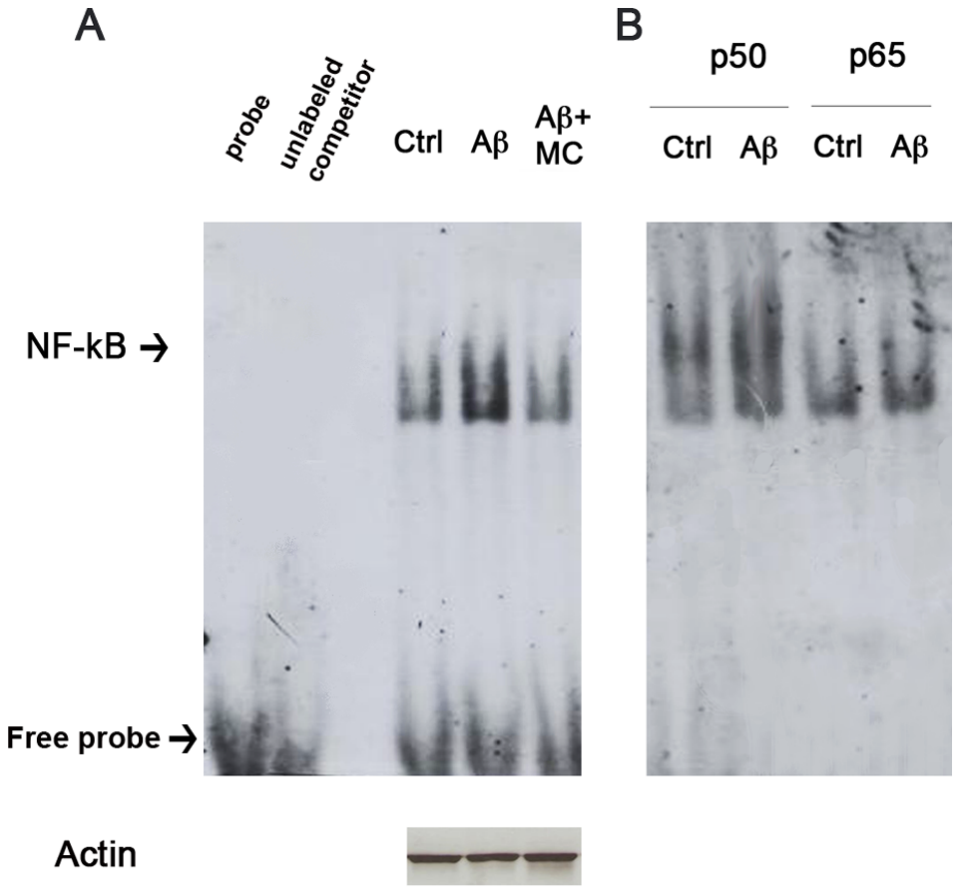
Aβ_25-35_ induces NF-kB activation *via* PARP. Cell lysates obtained from SH-SY5Y treated with 10 µM Aβ_25–35_ for 4 h, in the presence or absence of 50 µM MC2050, were incubated with a specific DNA probe for NF-kB, labeled with digoxigenin. A) The DNA-protein complexes separated on 4% native gels and transferred to nitrocellulose membrane were detected by chemiluminescence after hybridization with anti-DIG. B) Supershift of NF-kB detected by EMSA. The same cell lysates were incubated with the DNA probe for NF-kB in the presence of digoxigenin-labeled antibodies against p50 and p65 subunits.

Some authors have shown that Aβ can activate NF-kB in neuronal cells, suggesting that this molecular pathway may be responsible of the progression and of the pathogenesis of AD [26], [27]. Since Hassa and Hottinger [28] demostrated that PARP-1 regulates the function of NF-kB, we verified whether in our experimental conditions, Aβ_25–35_ modulates NF-kB activation through a PARP-1-dependent mechanism. Gel retardation assay (EMSA) showed a progressive increase of NF-kB activity during incubation with Aβ_25–35_. The o/n pre-treatment of SH-SY5Y cells with MC2050 followed by incubation with Aβ_25–35_ led to a significant reduction of NF-kB activation at 4 h (Fig. 6 A). We obtained similar results when we used PJ34 a commercially available PARP-1 inhibitor (data not shown).

To clarify which NF-kB subunit is responsible of the DNA binding activity, the cell extracts pre-treated or not with MC2050 were incubated with Aβ_25–35_ and antibodies against either p50 or p65. Figure 6B shows a slower electrophoretic migration of the complex in the presence of anti-p50, indicating the involvement of the p50 subunit in targeting the specific DNA sequence.

### MC2050 prevents NF-kB translocation from cytoplasm to nucleus in SH-SY5Y

Several authors have shown that poly (ADP-ribosylation) plays a role as an intracellular signal involved in the transport of transcription factors across the nuclear envelope [29], [30]. To verify whether PARP-1 plays a role in the nuclear translocation of NF-kB, the cytosolic and nuclear lysates, obtained from SH-SY5Y cells incubated with Aβ_25–35_ and pretreated in the presence or absence of MC2050 were analyzed by Western blot.

**Figure 7:**
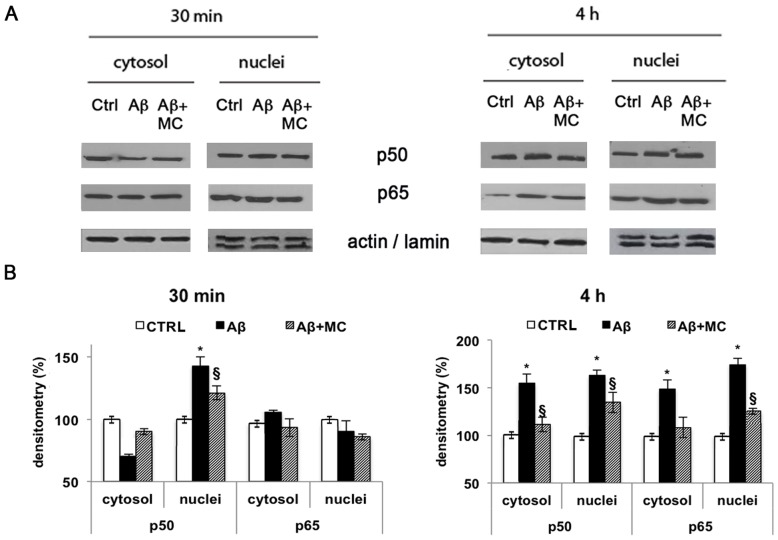
Effect of PARP-1 on NF-kB translocation and protein levels in SH-SY5Y treated with Aβ_25-35_. A) Cell lysates obtained at 30 min and 4 h from SH-SY5Y cells treated with 10 µM Aβ_25–35_ in the presence or absence of 50 µM MC2050, were for subjected to 12% SDS-PAGE, and immunoblotted with antibodies against p50 and p65. Proteins were detected through ECL (Millipore). B) Densitometric analyses were performed with ImageJ software, β-actin and lamin A/C levels were used as cytosolic and nuclear protein loading controls, respectively (white bar  =  control cells; black bar  =  Aβ_25–35_ treated cells; stripped bar  =  cells treated with Aβ_25-35_ in the presence of MC2050). Results are the mean of at least three independent experiments ± S.D. *p<0.05 *vs*. control cells; ^§^ *p<*0.05 *vs*. Aβ_25–35_-treated cells.

The results show that p50 subunit translocated in the nuclei after 30 min of treatment, on the contrary p65 localized in the nuclei after 4 h of treatment. We observed a 50% increase in both protein levels in the cytosol and nuclei within 4 h compared to control ones (Fig. 7 A, B). MC2050 pre-treatment induced a decrease of 20% and 50% in protein level of p50 and p65 subunits at 4 h, respectively.

### Aβ_25–35_ regulates p53 and Bcl-2 protein levels in mice and in SH-SY5Y

**Figure 8:**
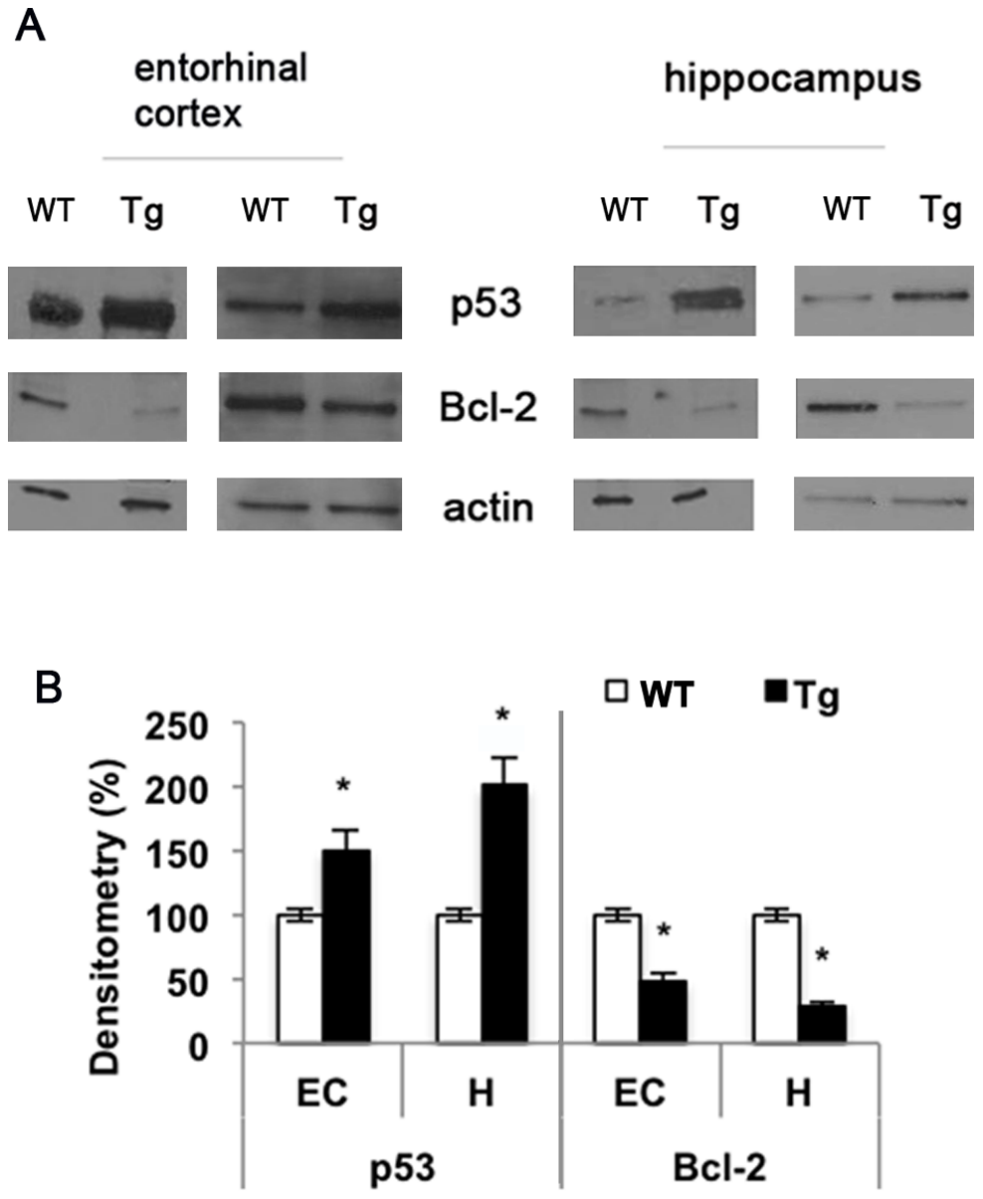
Aβ affects p53 and Bcl-2 protein levels in transgenic mice. A) Entorhinal cortex and hippocampus homogenates were subjected to 12% SDS-PAGE and immunoblotted with antibodies against p53 and Bcl-2. Proteins were detected through ECL (Millipore). B) Densitometric analysis was performed with ImageJ software and normalized to β-actin (white bar  =  Wild Type mice; black bar  =  Transgenic mice). Results are the mean of at least three independent experiments ± S.D. *p<0.05 *vs*. WT.

**Figure 9:**
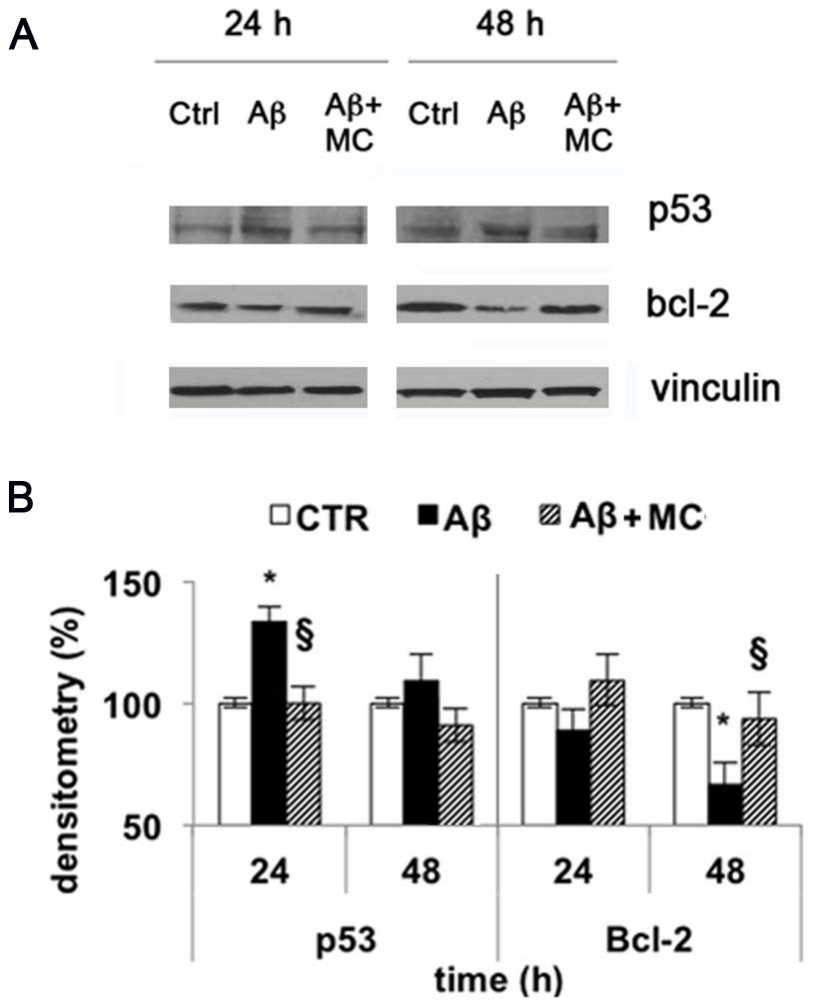
Aβ affects p53 and Bcl-2 protein levels in SH-SY5Y *via* PARP-1. A) Cells treated with 10 µM Aβ_25-35_ for 24 and 48 h were lysed, subjected to 12% SDS-PAGE and immunoblotted with antibodies against p53 and Bcl-2. Proteins were detected through ECL (Millipore). B) Densitometric analysis performed with ImageJ software, and normalized to vinculin (white bar  =  control cells; black bar  =  Aβ_25–35_ treated cells; stripped bar  =  cells treated with Aβ_25–35_ in the presence of MC2050). Results are the mean of at least three independent experiments ± S.D. *p<0.05 *vs*. control;^ §^ *p*<0.05 *vs*. Aβ_25–35_-treated cells.

In TgCRND8 mice, p53 protein levels increased by about 50% in the entorhinal cortex and 100% in hippocampus, compared to wild type mice, while the anti-apoptotic protein Bcl-2 decreased by about 50% and 75% in the same regions, respectively (Fig. 8 A, B). Thus, we assayed the effect of the amyloid peptide on these two apoptosis-related proteins in SH-SY5Y cell. Our data indicate that p53 protein levels increased by about 35% at 24 h while Bcl-2 levels decreased by about 30% within 48 h in cells treated with amyloid peptide compared to control. The pre-treatment of cells with the PARP-1 inhibitor MC2050 counteracted the apoptotic effect of Aβ_25–35_, restoring the basal protein levels (Fig. 9 A, B).

## Discussion

PARP-1 is the most abundant member of the family of PARP enzymes and plays a critical role in the maintenance of DNA integrity by signalling DNA damage to the DNA repair machinery [31], [32]. When high levels of DNA damage occur, such as during oxidative stress insult, PARP-1 activity increases considerably causing a drastic reduction of NAD^+^ levels, with consequences on the ATP production and impairment of cell functions [33], [34]. Several studies have shown that pharmacological inhibition or gene deletion of PARP-1 could prevent tissue damage induced by oxidative stress associated with several diseases like stroke, diabetes, myocardial ischemia-reperfusion and Parkinson's disease [35]–[37].

In patients affected by AD, Love et al. [16] observed high levels of poly (ADP-ribosylated) proteins in the brain and recently two groups reported high level of PARP-1 activity in astrocytes and hippocampal brain slices [38], [39]. Since oxidative stress induced by the amyloid peptide, is implicated in the pathogenesis of AD [40], [41] here we explored how the crosstalk between PARP-1, ROS and DNA damage contributes to the neurodegenerative phenotype in AD.

To reach our goal, SH-SY5Y cells incubated with the fragment Aβ_25–35_, in the presence or absence of a new PARP inhibitor, a 4-quinazoline derivative, MC2050 and 7PA2 cells overproducing Aβ_1–42_ were used as cellular models. Experiments were also carried out on transgenic mice carrying the double mutant form APP695, showing amyloid peptide deposition at 3 months of age.

Our results showed an increase of about 60% of the enzyme activity over 24 h in the treated SH-SY5Y cells using Aβ_25–35,_ a 40% increase in 7PA2 cells and 30% in hippocampal region of transgenic mice. The high level of PARylated proteins confirmed PARP-1 activation both in mice and in cells. The similar effect elicited by the two amyloidogenic species, confirms that the Aβ_25–35_ fragment is able to mimic the toxicity of Aβ_1–40/42_ and therefore is widely used to determine patterns of neurodegeneration. Millucci et al. [42] and Kaminsky et al. [43] reported the presence of the Aβ_25–35_ fragment in AD affected brains as the result of proteolysis of Aβ_1–40_ consequent to the racemization of serine 26. In comparative studies, Frozza et al. and Abramov et al. [44], [45] have shown that both Aβ_1–42_ and Aβ_25–35_ induce the same neuronal injury and toxic effects and Olivieri et al. [46] demonstrated the same effects on SH-SY5Y. Here we demonstrate that Aβ_25–35_ is able to induce PARP-1 activation in a similar manner to that reported for Aβ_1–40/42_ [31], [47]. Hence, all these researches support the use of Aβ_25–35_ as a suitable tool to study Alzheimer-type neurodegeneration.

The onset and development of AD are strictly associated with membrane integrity of neural cell. Indeed, oligomers and larger fibrillar aggregates made up by amyloid peptides seem to alter the biological function of the adjacent cellular membrane [24],[25]. Hence, first of all we assessed the ability of Aβ_25–35_ to interact with SH-SY5Y cell membrane. Data indicated that the Aβ_25–35_ targeted to cellular membrane inducing a 45% membrane perturbation after 4 h. An apparent decrease of membrane permeability was observed at 24 h, even if this phenomenon could be ascribed to cellular death. The neurotoxicity of Aβ_25–35_, assessed by the MTT assay, showed that the viability of cells treated with the amyloid peptide decrease of about 30% at 24 h, with a time-dependent trend, in agreement with the observations previously reported [48].

Many reports emphasized that in cells exposed to genotoxic insults, such as H_2_O_2_, PARP-1 is activated and modulates cell death [49], [50]. As shown by the cytofluorimetric analysis, Aβ_25–35_ induced oxidative stress and DNA damage as evaluated by comet assay that showed high DNA fragmentation in cells incubated with Aβ_25–35_ for 48 h. Then DNA damage led to PARP-1 activation that can be considered as one of the major cause of cell death probably depending on NAD depletion altering essential metabolic pathways as suggested by Abeti et al. in isolated astrocytes and in co-colture with hippocampal neurons [51].

Recently it has also been shown that PARP-1 plays an important role in the control of gene expression [52]. In particular, several studies explored the role of PARP-1 on the transcriptional activity of NF-kB, but the results are still ambiguous. Various authors have shown that the activation of NF-kB by PARP-1 is not due to its catalytic action, but to the interaction of PARP-1 with the two subunits of NF-kB [28], [53]. Conversely, other reports suggest a direct role of poly (ADP-ribosylation) in the co-activation of NF-kB [30], [47].

It is also known that NF-kB activation is strongly induced Aβ_1–40_, as well as Aβ_1–42_ in primary neurons or NT2N neuronal preparations, promoting the nuclear translocation of the subunits p65 and p50 and the expression of apoptotic genes [27]. Our data support the hypothesis that the activation of NF-kB caused by Aβ_25–35_ is mediated by PARP-1. In fact, by EMSA we show an increase of NF-kB activation in SH-SY5Y cells treated with Aβ_25–35_, which is prevented when the cells are pre-incubated with the PARP inhibitor MC2050. In addition, the “supershift” assay indicates p50 more than p65 as a component of the complex. Our data emphasize the role of PARP-1 as a trans-activator of NF-kB in the cells treated with the amyloid peptide, in agreement with data obtained by Chiarugi and Moskowitz [47], who used microglia and astrocytes as experimental model. Moreover, the specific involvement of the p50 subunit was also observed in vitro by Chang et al. [53], who demonstrated that NF-kappaB-p50 DNA binding was dependent on the presence of β-NAD^+^, and inhibited by the presence of PARP inhibitors.

Since activation of NF-kB is followed by its translocation from cytosol to nuclei as reported by Gilmore [54] and Perkins [55], we analysed the cellular localization of NF-kB. In SH-SY5Y cells treated with Aβ_25–35_ an initial translocation of p50 protein from the cytosol to the nucleus occurs within 30 min, whereas it seems that the p65 subunit is not implicated in this process. This data, together with the EMSA results, suggest that the p50 subunit is strictly involved in Aβ signal transduction. This hypothesis is supported also by data of Patel et al. [56] who observed p50 translocation in rat neurons following Aβ_25–35_ treatment.

We also tested whether the increase in NF-kB activity corresponds to an increase in NF-kB protein levels. We observed that Aβ_25–35_ affected the levels of p50 and p65 subunits in SH-SY5Y cells both in the nuclei and in the cytosol. Indeed after 4 h of treatment the levels of the two subunits significantly increase both in the cytosol and in the nuclei compared to control cells, indicating a positive regulation of NF-kB in cells exposed to Aβ. Data obtained from SH-SY5Y cells incubated with Aβ_25–35_ and pre-treated with MC2050 showed a decrease in protein levels of both subunits p50 and p65, suggesting that the significant p65 reduction in the nuclei could also be ascribed to a lack of p65 PARylation. Indeed, the translocation of NF-kB can be also modulated by PARP-1 through the poly (ADP-ribosylation) of the p65 subunit. The modified protein can no longer interact with the exportin Crm1, being retained in the nucleus [29].

Genotoxic insult such as oxidative stress and hypoxia cause DNA damage and trigger p53 activity. Misregulation in p53 activity can lead to cancer development, as well as to cardiovascular diseases, metabolic disorders and neurodegeneration.

We observed a significant increase in p53 protein levels in entorhinal cortex and hippocampus of transgenic mice and in SH-SY5Y cells treated with Aβ_25–35_. The observation that p53 is induced in cortex and brain of TgCRND8 mice point out the possible involvement of transcriptional regulation mechanisms. As a matter of fact, it was demonstrated that APP processing modulates p53 transcription via the signaling involving nuclear translocation of the AICD (APP intracellular domain) fragment generated by APP cleavage. Since mutant APP (more prone to gamma- and beta-secretase processing) is over-expressed in TgCRND8 mice, we can hypothesize that increased AICD is responsible for p53 up-regulation [57].

It is also well known that p53 is degraded rapidly in a ubiquitination-dependent proteasomal pathway and that the elevation in p53 levels occurs through stabilization and prevention of degradation [58]. Amyloid peptide can impair the proteasome, whose activity was found to be lower in AD brains than in age-matched controls [59], hence the altered levels of p53 in our experimental model could be also due to proteasomal impairment.

Once activated, p53 undergoes a large number of post-translational modifications including poly (ADP-ribosylation) that can stabilize the protein [60]. Several reports suggest a close relationship between PARP-1 and p53 [61] [62]. In experimental models of MPTP-induced parkinsonism, an increased level of p53 sustained by its the poly (ADP-ribosylation) prones neurons to cell death [17]. In our experimental model the pre-treatment of cells with MC2050 is able to counteract the toxic effects of Aβ_25–35_ thus restoring the basal p53 protein level.

Since Bcl-2 and p53 participate in the modulation and execution of cell death and since the increased expression of Bcl-2 protects PC12 cells from cell death induced by Aβ [63], we analyzed Bcl-2 protein levels in transgenic mice and SH-SY5Y cells treated with Aβ_25–35_. Our data indicated that the Bcl-2 protein level decreased markedly in both experimental models and that the pre-treatment of cells with MC2050 nullified the effect of amyloid treatment. These data are in agreement with Paradis et al. [64] who have shown that Aβ_1–40_ and Aβ_1–42_ induced a progressive decrease of Bcl-2 with a corresponding increase of the anti-apoptotic protein Bax in neuron primary cultures.

Since gene regulation occurs at different levels it is possible that PARP-1 affects Bcl-2 expression both at transcriptional or post-transcriptional level [65]. Recently, Dutta et al. [66] have demonstrated that Bcl-2 may influence PARP activity both in vitro and in cancer cells by a direct interaction with PARP-1. The overall relationship between PARP-1 and Bcl-2 may depend on the various inner environment of different cell lines and on the pathological condition.

In conclusion, our data show that PARP-1 has a prominent role of in the molecular mechanisms induced by Aβ that lead to cell death and neurodegeneration, through activation of NF-kB signalling and the modulation of p53 and Bcl-2.
